# A genome‐wide screen identifies genes that suppress the accumulation of spontaneous mutations in young and aged yeast cells

**DOI:** 10.1111/acel.13084

**Published:** 2019-12-18

**Authors:** Daniele Novarina, Georges E. Janssens, Koen Bokern, Tim Schut, Noor C. van Oerle, Hinke G. Kazemier, Liesbeth M. Veenhoff, Michael Chang

**Affiliations:** ^1^ European Research Institute for the Biology of Ageing University Medical Center Groningen University of Groningen Groningen The Netherlands

**Keywords:** genome stability, high‐throughput screen, mutagenesis, mutation rate, replicative aging, yeast

## Abstract

To ensure proper transmission of genetic information, cells need to preserve and faithfully replicate their genome, and failure to do so leads to genome instability, a hallmark of both cancer and aging. Defects in genes involved in guarding genome stability cause several human progeroid syndromes, and an age‐dependent accumulation of mutations has been observed in different organisms, from yeast to mammals. However, it is unclear whether the spontaneous mutation rate changes during aging and whether specific pathways are important for genome maintenance in old cells. We developed a high‐throughput replica‐pinning approach to screen for genes important to suppress the accumulation of spontaneous mutations during yeast replicative aging. We found 13 known mutation suppression genes, and 31 genes that had no previous link to spontaneous mutagenesis, and all acted independently of age. Importantly, we identified *PEX19*, encoding an evolutionarily conserved peroxisome biogenesis factor, as an age‐specific mutation suppression gene. While wild‐type and *pex19Δ* young cells have similar spontaneous mutation rates, aged cells lacking *PEX19* display an elevated mutation rate. This finding suggests that functional peroxisomes may be important to preserve genome integrity specifically in old cells.

## Graphical abstract

We developed a high‐throughput approach to screen for genes important to suppress the accumulation of spontaneous mutations during yeast replicative aging. We found 13 known and 32 novel mutation suppression genes. Remarkably, one of these genes, *PEX19*, encoding an evolutionarily conserved peroxisome biogenesis factor, acts in an age‐dependent manner; while wild‐type and *pex19Δ* young cells have similar spontaneous mutation rates, aged cells lacking *PEX19* display an elevated mutation rate.

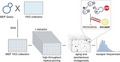

## 1 INTRODUCTION

Genomic instability, which refers to an increased rate of accumulation of mutations and other genomic alterations, is a hallmark, and a likely driving force, of tumorigenesis. Genomic instability is also a hallmark of aging (Maslov & Vijg, 2009), as suggested by the age‐related accumulation of mutations observed in yeast, flies, mice, and humans (Moskalev et al., 2013), and highlighted by the fact that defects in DNA repair pathways result in human premature aging diseases (Carrero, Soria‐Valles, & López‐Otín, 2016). However, whether genome instability has a causative role in aging is still controversial (Moskalev et al., 2013), and it is not known whether aged cells rely more heavily on specific genome maintenance pathways.

*Saccharomyces cerevisiae* is a convenient model to study genomic instability and its relationship with aging since genome maintenance pathways are evolutionarily conserved (Putnam & Kolodner, 2017; Putnam et al., 2016) and a large number of genetic assays have been developed to study DNA repair and mutagenesis in budding yeast (Putnam & Kolodner, 2017). Furthermore, most aging‐related cellular pathways, as well as lifespan‐modulating environmental and genetic interventions, show a remarkable degree of conservation from yeast to mammals (Kaeberlein, 2010). There are two main *S. cerevisiae* aging models: Replicative aging refers to the decline in viability that a cell experiences with increasing number of mitotic divisions (a model for aging of mitotically active cells), while chronological aging refers to the decline in viability of a nondividing cell as a function of time (a model for aging of postmitotic cells; Kaeberlein, 2010).

To identify genes and pathways involved in mutagenesis during yeast replicative aging, two main challenges need to be overcome. First, while *S. cerevisiae* is a leading model system for genetic and genomic studies and the availability of the yeast deletion collection makes this model organism particularly amenable for genome‐wide genetic screens, cellular processes involving low‐frequency events, such as point mutations, recombination events, or gross chromosomal rearrangements, pose a specific technical challenge, since these events are barely detectable with standard genome‐wide screening methods. In a pioneering study, Huang and colleagues performed a genome‐wide screen for yeast genes that suppress the accumulation of spontaneous mutations in young cells by screening patches of large numbers of cells on solid media (Huang, Rio, Nicolas, & Kolodner, 2003). Patches of each strain of the deletion collection were replica‐plated on media containing canavanine to detect canavanine‐resistant (Can^R^) colonies arising from spontaneous mutations at the *CAN1* locus. A similar approach was subsequently used in other screens for genes controlling genome integrity (Putnam et al., 2016; Smith et al., 2004; Zhang et al., 2012). This strategy has proven to be effective but is extremely laborious. To overcome these limitations, we developed a screening strategy to detect low‐frequency events, based on high‐throughput replica pinning of high‐density arrays of yeast colonies.

**Figure 1:**
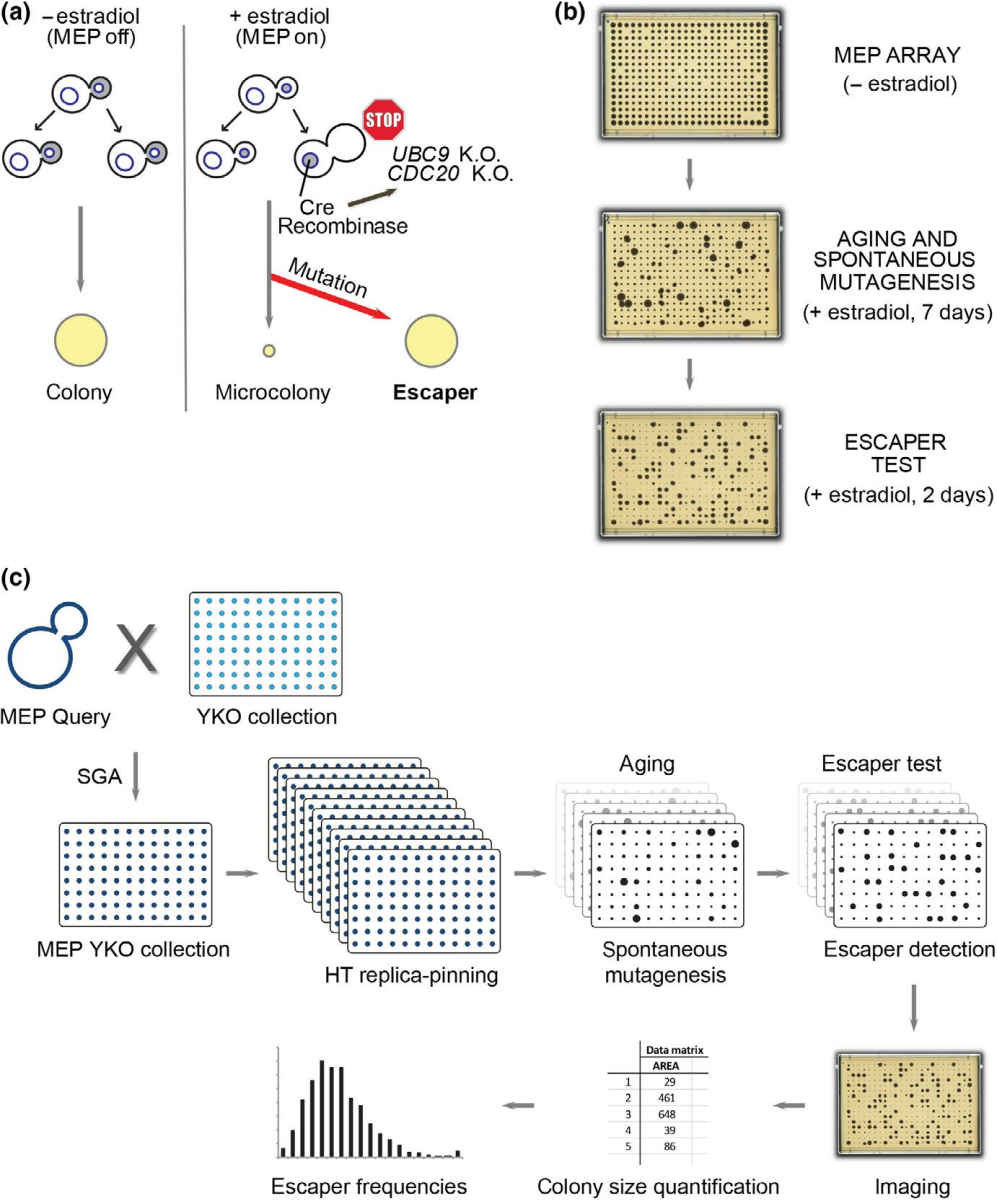
Combining the Mother Enrichment Program with high‐throughput replica pinning to screen for genes that suppress spontaneous mutations during yeast replicative aging. (a) The Mother Enrichment Program (MEP). Estradiol induction causes irreversible arrest of daughter cell proliferation, while growth of mother cells is unaffected. Inactivation of the MEP due to spontaneous mutations results in estradiol‐insensitive cells called escapers. (b) Escaper formation is a readout for spontaneous mutation events during replicative aging. High‐density arrays of MEP colonies are pinned on estradiol, and escapers are subsequently detected by re‐pinning on estradiol (big colonies). (c) Schematic of the screening procedure. See main text for details

The second challenge to identify genes involved in mutagenesis during yeast replicative aging is the isolation of aged cells, since they constitute a tiny fraction of an exponentially growing cell population. To allow the study of a cohort of aging mother cells, Lindstrom and Gottschling developed the Mother Enrichment Program (MEP), an inducible genetic system that prevents the proliferation of daughter cells (Figure 1a; Lindstrom & Gottschling, 2009). Upon activation by estradiol, the Cre recombinase, which is under the control of a daughter‐specific promoter, enters the nucleus and disrupts two genes essential for cell cycle progression (namely *UBC9* and *CDC20*), resulting in an irreversible arrest of daughter cells in G2/M, while mother cells are unaffected. Thus, in the absence of estradiol, MEP cells grow exponentially and form normal colonies on an agar plate, while upon addition of estradiol, linear growth occurs and microcolonies are formed. Occasionally, due to spontaneous mutations, the MEP is inactivated and cells become insensitive to estradiol: These cells are called “escapers” (Lindstrom & Gottschling, 2009). Escaper cells grow exponentially and form normal‐sized colonies even in the presence of estradiol.

We combined the MEP system with a high‐throughput replica‐pinning strategy to perform a genome‐wide screen aimed at identifying genes important to suppress the accumulation of spontaneous mutations in replicatively aging yeast cells, using escaper formation as a readout for spontaneous mutagenesis events. With our approach, we identified several new mutation suppression genes that act independently of age. We also found that *PEX19*, involved in peroxisome biogenesis, is an age‐specific mutation suppression gene: While wild‐type and *pex19Δ* young cells have similar spontaneous mutation rates, the absence of *PEX19* causes an elevated mutation rate specifically in old cells.

## 2 RESULTS

### 2.1 A high‐throughput screen to identify genes important for suppressing spontaneous mutations during yeast replicative aging

We developed a high‐throughput replica‐pinning strategy that enables the detection of low‐frequency events and used it to perform a genome‐wide screen for genes important for suppressing spontaneous mutations during yeast replicative aging (Figure 1). We introduced the MEP system into the yeast knockout (YKO) collection via Synthetic Genetic Array (SGA) technology (Tong & Boone, 2006). The resulting MEP‐YKO collection was pinned multiple times in parallel on estradiol‐containing plates (18 replicates per knockout strain) to activate the MEP and grown for 1 week (Figure 1c). An example of our experimental setting is shown in Figure 1b. If at any time during aging a MEP‐inactivating mutation occurs, an escaper colony is formed. Each plate was then re‐pinned on estradiol and grown for 2 days to detect escapers (escaper test). At this point, all MEP‐proficient mother cells that have exhausted their replicative potential are not able to give rise to a colony; conversely, if escaper cells are present, a fully grown colony can be observed (Figure 1b,c). Our high‐throughput replica‐pinning method allows the semi‐quantitative estimation of spontaneous mutation rates on the basis of escaper formation (Figure 1c). Since every plate of the MEP‐YKO collection was pinned multiple times in parallel on estradiol, it is possible to calculate the frequency of escaper formation for each deletion mutant strain in the collection: An increased escaper frequency compared with wild‐type control strains is an indication of a high spontaneous mutation rate.

**Figure 2:**
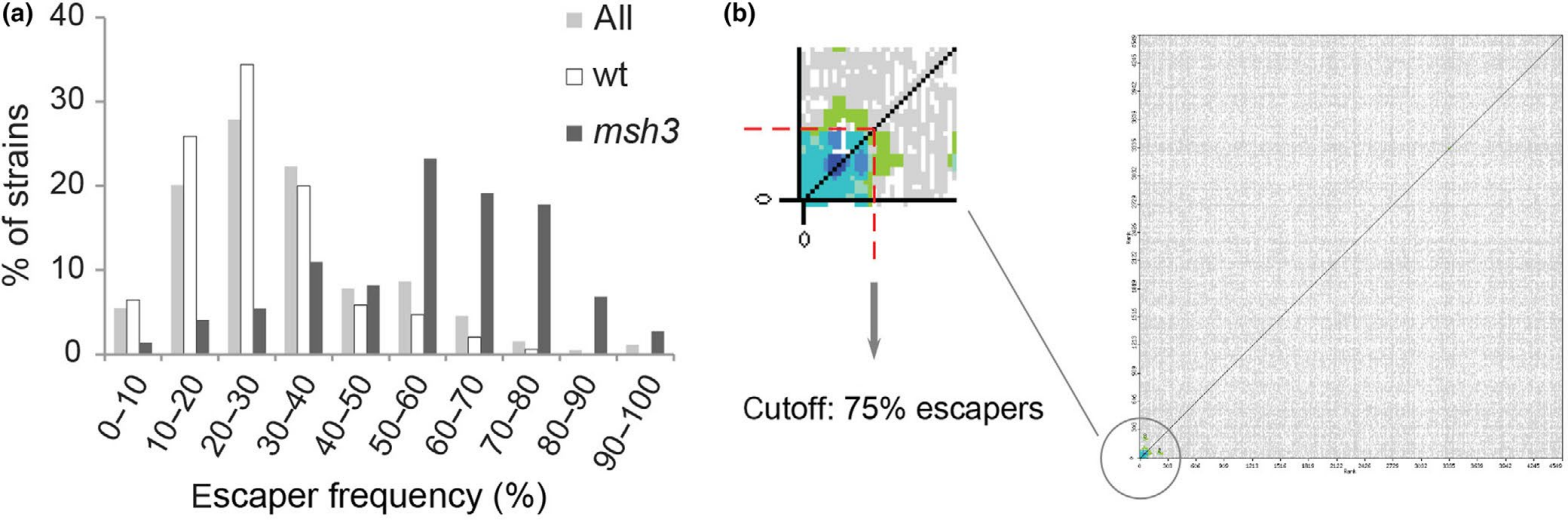
Analysis of the screen. (a) Comparison of escaper frequencies of the whole MEP‐YKO collection with escaper frequencies of wt and mutator (*msh3*) controls. (b) CLIK analysis sets an unbiased cutoff for validation. Green and blue colors indicate regions of the plot significantly enriched for physical and genetic interactions, while regions deprived of significant enrichment are plotted in gray. The red dotted line marks the cutoff suggested by the CLIK algorithm

To validate our assumption that the escaper frequency of each strain is a proxy for the spontaneous mutation rate, we could make use of the fact that 72 strains from the YKO collection were derived from a parental strain carrying an additional mutation in the mismatch repair gene *MSH3* and are therefore expected to show increased spontaneous mutation rates, independently of the identity of the knockout gene (Lehner, Stone, Farber, & Petes, 2007). In addition, 340 empty positions randomly dispersed over the 14 plates of the MEP‐YKO library were manually filled in with a wild‐type MEP control. In Figure 2a, an overview of the escaper frequencies of the whole MEP‐YKO collection is shown. Most of the strains have an escaper frequency between 10% and 40% (median: 27.8%). The wild‐type control strains show a similar behavior (median: 22.2%), but with the important difference that the wild‐type escaper frequency never exceeds 72.2%. In contrast, the escaper frequency of most of the *msh3* strains falls between 50% and 80% (median: 55.6%), validating the rationale of our screening method.

We then used Cutoff Linked to Interaction Knowledge (CLIK) analysis (Dittmar, Pierce, Rothstein, & Reid, 2013) to determine the cutoff for validation in an unbiased manner. The CLIK algorithm identified an enrichment of highly interacting genes at the top of our list (ranked according to escaper frequency), confirming the overall high quality of our screen (Figure 2b). The cutoff suggested by CLIK corresponds to an escaper frequency of 75%, which, not surprisingly, is slightly higher than the maximum escaper frequency observed in the wild‐type controls (72.2%). To further explore the overall quality of the screen, we set the cutoff at 75% escaper frequency and performed phenotypic enrichment analysis using ScreenTroll, which examines the similarity between genome‐scale screens (Thorpe, Dittmar, & Rothstein, 2012). Predictably, the first overlap with our gene list is the mutator screen performed by Huang et al. (2003). Furthermore, most of the top overlapping screens are related to genome instability and DNA damage sensitivity (Table S1). Based on the threshold determined by CLIK, we proceeded to direct validation of all hits with an escaper frequency higher than 75%.

### 2.2 Identification of new genes that suppress the accumulation of mutations independently of replicative age

We first discarded as false positive all hits where the escaper‐causing mutation(s) had occurred before the beginning of the aging experiment (i.e., during the generation of the MEP‐YKO library and before the subsequent high‐throughput replica‐pinning step), as in these cases, an escaper frequency of 100% is not an indication of an extremely high mutation rate. By spotting serial dilutions of strains from the MEP‐YKO library on estradiol‐containing plates (Figure S1), we found that 25/115 hits had escaped before the actual screen started (File S1). We then set out to validate the remaining 90 putative mutator strains.

**Figure 3:**
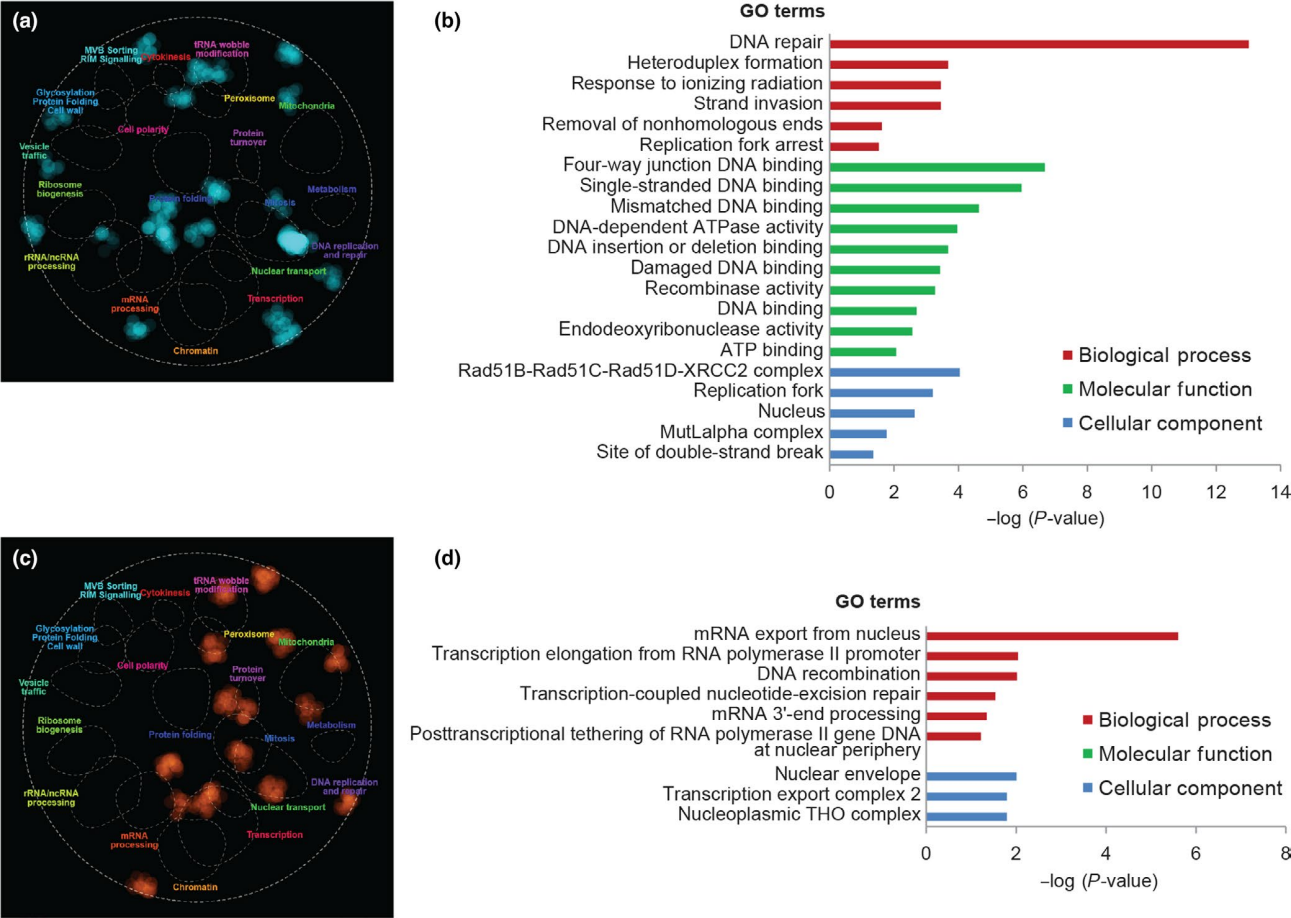
Overview of the general and MEP‐specific mutation suppression genes. (a, c) Functional enrichment in the yeast genetic landscape. Dotted lines indicate functional domains within the yeast genetic landscape, that is, gene clusters enriched for a specific set of GO terms (the name of each functional domain is indicated by a colored label). Regions of the global similarity network significantly enriched for general (a) or MEP‐specific (c) mutation suppression genes were mapped using SAFE (Baryshnikova, 2016) and are indicated in blue and orange, respectively. (b, d) Gene Ontology enrichment analysis of general (b) and MEP‐specific (d) mutation suppression genes

Spontaneous mutations can occur at any moment of the replicative lifespan, and our experimental design does not allow us to discriminate whether a high escaper frequency is an indication of an increased mutation rate already in young cells, or of an elevated age‐dependent accumulation of mutations. To distinguish between these two possibilities, we performed fluctuation tests to measure the forward mutation rate (expressed as number of mutations per cell division) at the endogenous *CAN1* locus, where any type of mutation that inactivates the *CAN1* gene confers canavanine resistance (Foster, 2006). Since fluctuation tests are performed with logarithmically growing cultures, they measure the spontaneous mutation rate in an age‐independent fashion (i.e., in young cells). Twelve of our hits had been previously validated in the same BY4741 strain background used for our validations (Huang et al., 2003) and therefore were not retested. Several genes identified in the aforementioned study (namely *CSM2*, *SHU1*, *TSA1*, and *SKN7*) fell just below our 75% escaper frequency cutoff (File S1). The deletion of *MPH1* has also been reported to increase spontaneous mutation rates of *CAN1* (Scheller, Schürer, Rudolph, Hettwer, & Kramer, 2000), but since this study was performed in a different strain background, we retested the phenotype in the BY4741 background. Importantly, we validated 13 new mutator mutants. Our screening strategy thus enabled us to identify new genes important for the suppression of spontaneous mutations independently of replicative age. The 26 genes (12 from Huang et al., *MPH1*, and the 13 newly identified genes) whose deletion results in an increased *CAN1* mutation rate of at least 1.8‐fold compared with wild‐type are listed in Table 1. We named this group of genes “general mutation suppression genes” because the corresponding knockout strains, besides showing an elevated escaper frequency in our screening setup, also display an increased mutation rate when tested in young cells with a second assay for spontaneous mutagenesis at a different genetic locus. Of the general mutation suppression genes identified, 16/26 have one or more human orthologs. As expected, these genes are significantly enriched for Gene Ontology categories related to DNA damage response, DNA repair, and recombination (Figure 3,b, and File S2).

**Table 1:** List of validated general mutation suppression genes

	Gene deleted	Can^R^ rate (× 10^–7^)	Function	Human ortholog(s)^a^
1	*RAD27*	167.1^b^	DNA replication and repair	*FEN1*,* GEN1*
2	*PMS1*	79.9^b^	Mismatch repair	*PMS1*,* PMS2*
3	*MSH2*	64.9^b^	Mismatch repair	*MSH2*
4	*MLH1*	53.0^b^	Mismatch repair	*MLH1*
5	*MME1*	45.1^b^	Magnesium ion export from mitochondrion	
6	*RAD54*	37.9^b^	Recombinational repair	*ATRX*,* RAD54B*,* RAD54L*,* RAD54L2*
7	*RAD57*	37.2^b^	Recombinational repair	*XRCC3*
8	*RAD55*	35.8^b^	Recombinational repair	
9	*MPH1*	29.5^c^	Error‐free bypass of DNA lesions	*FANCM*
10	*MSH6*	27.7^b^	Mismatch repair	*MSH6*
11	***YGL177W***	27.6	Dubious open reading frame (overlaps *MPT5*)	
12	***CRS5***	23.6	Copper‐binding metallothionein	
13	***VMA6***	22.5	V‐ATPase	*ATP6V0D1*,* ATP6V0D2*
14	***GRX7***	21.7	Oxidative stress response	
15	*PSY3*	20.4^b^	Error‐free DNA lesion bypass	
16	*OGG1*	17.6^b^	DNA repair	*OGG1*
17	*SHU2*	17.4^b^	Error‐free DNA lesion bypass	
18	***NAT3***	17.0	NatB *N*‐terminal acetyltransferase	*NAA20*
19	***APL1***	16.3	Vesicle‐mediated transport	
20	***MET18***	8.1	Fe‐S cluster assembly	*MMS19*
21	***YLR358C***	8.0	Unknown (overlaps *RSC2*)	
22	***DSS4***	5.4	Post‐Golgi vesicle‐mediated transport	
23	***RLF2***	5.2	Chromatin assembly complex	*CHAF1A*
24	***SRP40***	5.0	Preribosome assembly or transport	*NOLC1*
25	***NUP84***	4.9	Nuclear pore complex	*NUP107*
26	***RAD10***	4.9	DNA repair	*ERCC1*
	Wild‐type	2.6		

Note

Genes in bold were newly identified; genes in regular font were previously identified (Huang et al., 2003; Scheller et al., 2000).

a Human orthologs of yeast genes are taken from the “*S. cerevisiae* to human ortholog pairs” tool from the Rothstein Lab website (http://www.rothsteinlab.com).

b Values from Huang et al. (2003); all other values were determined as described in the Materials and Methods and are statistically supported (Student's *t* test).

c An elevated mutation rate for this mutant has been previously reported (Scheller et al., 2000).

Many genes are annotated as suppressors of mutations in the *Saccharomyces* Genome Database (phenotype: mutation frequency: increased), although most of these annotations are not referring to spontaneous mutagenesis at the *CAN1* locus. Among this list, our screen only identified those found in the Huang et al. study, plus *MPH1*. If we assume that the 31 genes from the Huang et al. study and *MPH1* (i.e., 32 genes in total) encompass all previously known *CAN1* mutation suppression genes, then we identified 41% (13/32) of these genes. This percentage would likely be even higher if the original assay in our screen was based on *CAN1* mutagenesis, and if we had used a less stringent cutoff (as noted above, four genes identified in the Huang et al. study fell just below our cutoff). Our 13 newly identified genes bring the total known *CAN1* mutation suppression genes to 45 (32 + 13), and we estimate that approximately 19 *CAN1* mutation suppression genes remain to be identified [(26/0.41)–45].

We then determined whether the 64 strains (90 tested minus the validated 26) that do not show an elevated mutation rate at the *CAN1* locus would display an increase in the mutation rate if measured by escaper formation in the MEP genetic background. To do so, we performed a slightly modified version of the fluctuation test, where selective (i.e., estradiol‐containing) plates are incubated for 7 days and colonies are counted after 2 and 7 days. Escaper colonies appearing after 2 days of incubation originate from mutations occurring prior to plating (i.e., in young cells), while all colonies appearing between day 2 and day 7 originate from a mutation event that occurred during replicative aging (see Experimental Procedures for details). This experimental setup mimics the conditions in which the initial screen was performed and allows us to simultaneously measure the escaper formation rates in young cells and the age‐dependent escaper formation frequencies. With this assay, we identified 18 genes whose deletion results in an increased escaper formation rate of at least 1.8‐fold compared with the wild‐type, independently of replicative age (i.e., based on colonies counted at day 2). We named these genes “MEP‐specific mutation suppression genes,” since the spontaneous mutation rate measured at the *CAN1* locus in the corresponding knockout mutants is indistinguishable from the wild‐type (Table 2). About half (8/18) of these genes have one or more human orthologs. Intriguingly, MEP‐specific mutation suppression genes are enriched for members of the THO/TREX complex, which is involved in co‐transcriptional mRNA export from the nucleus. This process is important in the interplay between transcriptional elongation and R‐loop formation (Huertas & Aguilera, 2003; Figure 3c,d, and File S2).

**Table 2:** List of validated MEP‐specific mutation suppression genes

	Gene deleted	Escaper rate (× 10^–7^)^a^	Function	Human ortholog(s)^a^
1	*XRN1*	84.1	Exoribonuclease	*XRN1*
2	*COS10*	54.6	Turnover of plasma membrane proteins	
3	*NIP100*	52.1	Dynactin complex	*CEP350*,* CLIP1*,* CLIP2*,* CLIP3*,* CLIP4*
4	*RPP1A*	34.4	Ribosomal protein	*RPLP1*
5	*FMP45*	29.7	Mitochondrial membrane protein	
6	*RPL13B*	26.3	Ribosomal protein	*RPL13*
7	*HOP2*	25.2	Meiosis	
8	*SAC3*	17.1	mRNA export (TREX complex)	*MCM3AP*,* SAC3D1*
9	*HBT1*	14.2	Polarized cell morphogenesis	
10	*NFT1*	12.2	Putative ABC transporter	
11	*SNF2*	7.9	SWI/SNF chromatin remodeling complex	*SMARCA2*,* SMARCA4*
12	*MFT1*	7.6	mRNA export (THO complex)	
13	*GTO3*	7.6	Glutathione transferase	
14	*THP1*	7.2	mRNA export (TREX complex)	
15	*SEM1*	6.1	mRNA export/proteasome regulation	*SHFM1*
16	*YPL205C*	6.0	Dubious open reading frame	
17	*THP2*	5.9	mRNA export (THO/TREX complex)	
18	*UBA4*	5.6	Thio‐modification of tRNA	*MOCS3*,* UBA5*
	Wild‐type	2.9		

All values shown are statistically supported (Student's *t* test).

a Human orthologs of yeast genes are taken from the “*S. cerevisiae* to human ortholog pairs” tool from the Rothstein Lab website (http://www.rothsteinlab.com).

### 2.3 *PEX19* suppresses age‐dependent accumulation of mutations

**Figure 4:**
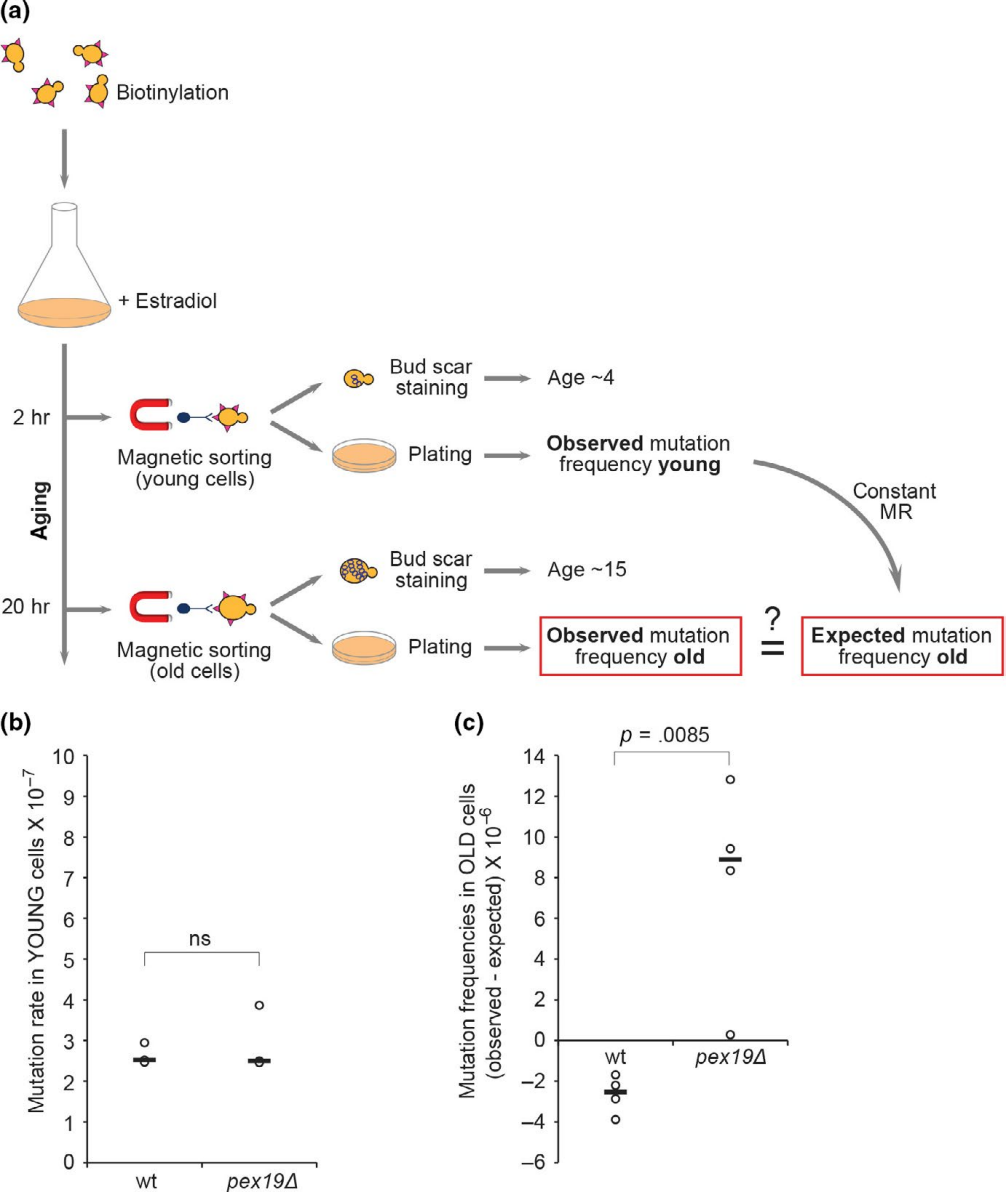
*PEX19* suppresses age‐dependent accumulation of mutations. (a) Schematic of the experiment to measure age‐dependent mutation frequency. See main text for details. (b) *CAN1* forward mutation rate in young wt and *pex19∆* cells. Values from three independent experiments are plotted. The thick dark bars represent the median values. ns: nonsignificant. (c) Age‐dependent mutation frequencies at the *CAN1* locus in wt (replicative age ~17) and *pex19∆* (replicative age ~15.5) cells. The difference between observed and expected mutation frequencies from four independent experiments is plotted. The horizontal bars represent the median values. *p*‐value was determined by Student's *t* test

At the end of our validation pipeline, we were left with four gene knockout strains that display no significant increase in forward mutation rate at the *CAN1* locus and in escaper formation rate in young cells (colonies counted at day 2) but show a higher age‐dependent escaper frequency compared with wild‐type (colonies counted at day 7; Table S2). We were particularly interested in these genes, since our observations might indicate an age‐dependent mutator phenotype. To validate these putative age‐specific mutator mutants with an independent and more accurate method, we mechanically isolated young and aged mother cells by biotinylation and magnetic sorting and measured mutation frequencies at the *CAN1* locus in both cell populations (Patterson & Maxwell, 2014; Figure 4a). Based on the Can^R^ frequencies in young cells, the replicative age (assessed by bud scar counting), and the mutation rate in young cells (previously determined by fluctuation test), we could calculate the expected Can^R^ frequencies in aged cells under the assumption that the mutation rate remains constant during replicative aging (see Experimental Procedures for details). By comparing the observed and the expected frequencies, it becomes clear whether the mutation rate of a given strain is constant or varies as cells age.

To establish a reference, we tested wild‐type cells. Strikingly, the observed mutation frequency in aged cells (replicative age ~17) was lower than expected (Figures 4c and S2), suggesting a decrease in the spontaneous mutation rate during replicative aging. We then measured mutation frequencies in young and old cells from the four putative age‐specific mutator strains. After the first test, three of these strains did not show any increase in age‐dependent mutation frequency compared with the wild‐type and were therefore discarded as false positives (Figures S3 and S2). Conversely, age‐dependent mutation frequency in the absence of *PEX19* was higher than in the wild‐type. We therefore repeated the test and confirmed that *pex19∆* aged cells (replicative age ~15.5) display much higher mutation frequencies than expected (Figure 4c; File S2). This result suggests that *PEX19*, encoding an evolutionarily conserved factor required for peroxisome biogenesis (Hettema, Girzalsky, Berg, Erdmann, & Distel, 2000; Jansen & van der Klei, 2019), suppresses age‐dependent accumulation of mutations. We observed a similar effect after the deletion of *PEX3* (Figure S4), which causes the same peroxisome biogenesis defect as observed in the absence of *PEX19*, namely lack of detectable peroxisomal structures (Hettema et al., 2000).

Peroxisomes are key organelles for the maintenance of the redox balance of the cell. On the one hand, they generate hydrogen peroxide as a consequence of fatty acid peroxidation; on the other hand, they contain a set of antioxidant enzymes and function therefore as reactive oxygen species scavenging organelles (Bonekamp, Völkl, Fahimi, & Schrader, 2009). Cells lacking Pex19 may have higher levels of oxidative damage, including DNA lesions. These lesions may be efficiently repaired in young cells but not in aged cells, where DNA repair pathways may be compromised, leading to an age‐dependent accumulation of mutations. If true, *pex19∆* cells may become increasingly sensitive to exposure to oxidative or genotoxic agents as they age. However, we found that aged *pex19∆* cells are not more sensitive to hydrogen peroxide or the DNA damaging agent methyl methanesulfonate than young *pex19∆* cells (Figure S5).

## 3 DISCUSSION

*Saccharomyces cerevisiae* is an outstanding model in which to perform genetic screens. However, there has been a lack of genome‐wide, high‐throughput screening techniques to detect low‐frequency events (such as point mutations, recombination events, and gross chromosomal rearrangements). These technical limitations, in combination with the difficulty to isolate large populations of replicative old cells, have so far hindered the study of genome maintenance during replicative aging.

### 3.1 Potential applications of the high‐throughput replica‐pinning methodology

We developed a high‐throughput replica‐pinning approach to screen for cellular processes involving low‐frequency events, thus filling a technological gap in the yeast screening field. We applied this strategy to screen for genes controlling the accumulation of spontaneous mutations during yeast replicative aging, using the MEP both as a tool to induce replicative aging and as a reporter for spontaneous mutation events (Figure 1). Key technical adjustments (see Experimental Procedures) were as follows: (a) the use of 1536 format pads to pin the MEP‐YKO collection in 384 format on estradiol, so that a smaller number of cells (~1.5 × 10^4^) was deposited on the plate, to prevent nutrient limitation during replicative aging; (b) the MEP‐YKO collection amplification by parallel high‐throughput replica pinning to analyze 18 colonies per strain; and (c) the 1‐week incubation time, which allowed the accumulation of spontaneous mutations throughout replicative lifespan. In this way, we were able to monitor enough cell divisions to detect low‐frequency mutation events. Furthermore, the analysis of 18 independent colonies allowed the use of escaper frequency as a proxy for the spontaneous mutation rate. Bioinformatic analysis and experimental confirmation indicated the high quality of the screen, thus validating our methodology.

One caveat of our screen approach for identifying mutant strains with an age‐dependent mutator phenotype is that strains with a short replicative lifespan will be underrepresented because they are more likely to die before escapers can form. Thus, there may be additional genes that specifically suppress the accumulation of mutations in aged cells. Importantly, this caveat would not apply to age‐independent mutation suppression genes; indeed, our screen identified many such genes that yield short replicative lifespans when deleted (i.e., *RAD27*, *RAD57*, *RAD55*, and *VMA6*; Table S5).

It is worth noting that, even independently of the MEP and the replicative aging perspective, a similar high‐throughput replica‐pinning approach can be used to screen for genes involved in other genome stability‐related processes. For instance, we recently applied this strategy to study spontaneous homologous recombination events (manuscript in preparation). Similarly, our replica‐pinning strategy could be adapted to screen for genes controlling genome integrity in the chronological lifespan model (Powers, Kaeberlein, Caldwell, Kennedy, & Fields, 2006). More generally, this technique can be used to study any process involving low‐frequency events for which genetically selectable reporters exist or can be developed. Examples include transient (loss of) gene silencing (Dodson & Rine, 2015), transcription errors (Gout et al., 2017), and read‐through at premature termination codons (Altamura et al., 2016).

### 3.2 Identification of novel age‐independent mutation suppression genes

Our screening setup was designed to allow simultaneous identification of age‐independent and age‐specific mutator mutants. We identified 13 new genes that suppress the accumulation of spontaneous mutations at the *CAN1* locus independently of age (Table 1). Some of these general mutation suppression genes (*RAD10* and *NUP84*) have defined roles in genome integrity (Bardwell, Bardwell, Tomkinson, & Friedberg, 1994; Freudenreich & Su, 2016). For some other well‐characterized genes, their role in preventing the accumulation of mutations can be inferred from their molecular function. For instance, *MET18* has a conserved role in iron–sulfur (Fe/S) cluster assembly and insertion in several proteins involved in DNA replication and repair (Stehling et al., 2012). *RLF2*/*CAC1* encodes the largest subunit of the chromatin assembly factor‐I complex, for which a function in DNA repair has been described (Game & Kaufman, 1999). The role of the other genes (*VMA6*, *GRX7*, *CRS5*, *NAT3*, *DSS4*, *APL1*, *YGL177W*, *YLR358C*, *SRP40*) in protecting the genome from spontaneous mutations might be more indirect and requires further investigation.

We also identified 18 mutants that, despite an undetectable increase in the spontaneous mutation rate at the *CAN1* locus, display an age‐independent elevated escaper formation rate (Table 2). This observation hints at a locus‐specific increase in mutagenesis for this group of mutator strains. The observation that MEP‐specific mutation suppression genes are enriched for genes involved in mRNA export from the nucleus (Figure 3; File S2) suggests the involvement of R‐loop‐dependent genome instability (Huertas & Aguilera, 2003). R loops form preferentially at specific genomic locations and can cause genomic instability by exposing single‐stranded DNA tracts, triggering hyper‐recombination and interfering with DNA replication (Huertas & Aguilera, 2003). Intriguingly, the exoribonuclease encoded by *XRN1*, our top MEP‐specific mutation suppression gene, has also been implicated in preventing R‐loop‐dependent genome instability (Wahba, Amon, Koshland, & Vuica‐Ross, 2011). To confirm this hypothesis, one would need to examine the genomic features of the locus or loci where mutations that give rise to escapers happen. It is assumed that escaper‐originating mutations occur at the *cre‐EBD78* locus (since inactivating the Cre recombinase results in a disruption of the MEP system), but the creators of the MEP already suggested that this is not always the case, and other unknown endogenous loci might be involved in escaper formation (Lindstrom & Gottschling, 2009). Indeed, our genetic analysis of a few escapers originating from a wild‐type MEP strain showed that the escaper phenotype does not always co‐segregate with the *cre‐EBD78* locus, confirming that mutations occurring at other genomic loci can result in MEP inactivation and escaper formation (Table S3).

### 3.3 A decrease in the spontaneous mutation rate in aged yeast cells

To investigate age‐dependent spontaneous mutagenesis, we first compared mutation frequencies at the *CAN1* locus in young and old wt cells. Interestingly, *CAN1* mutation frequencies in aged cells are lower than predicted, indicating that spontaneous mutation rates decrease during replicative aging (Figure 4c). This might occur, for instance, if the efficiency of a mutagenic DNA repair pathway, such as translesion synthesis (Boiteux & Jinks‐Robertson, 2013), is reduced in old cells. The same effect would be observed if an error‐free repair pathway is upregulated during aging. A similar decrease in the spontaneous mutation rate at the *CAN1* endogenous locus has been previously reported, although the same study suggested that this effect could be locus‐specific (Patterson & Maxwell, 2014).

### 3.4 A role for Pex19 in suppressing age‐dependent accumulation of spontaneous mutations

To better understand age‐dependent mutagenesis, our screen aimed at identifying genes—if they exist—that prevent accumulation of spontaneous mutations specifically in old cells. We show that *PEX19* is one of these genes, since its deletion has no effect on mutagenesis in young cells, but causes an elevated accumulation of mutations in aged cells (Figure 4). To our knowledge, this is the first described case of an age‐dependent mutation suppression gene, suggesting that some cellular pathways are particularly important in protecting the genome of old cells. *PEX19* is an evolutionarily conserved gene that plays a key role in peroxisome biogenesis, and whose absence results in the lack of detectable peroxisomes (Hettema et al., 2000; Jansen & van der Klei, 2019). The deletion of *PEX3*, another peroxisome biogenesis gene, causes the same age‐specific mutator phenotype (Figure S4), implying that functional peroxisomes are important to prevent age‐dependent accumulation of mutations. However, Pex19 and Pex3 have also been implicated in processes not directly related to peroxisomes, such as sorting of certain membrane proteins to mitochondria, lipid droplets, and the endoplasmic reticulum (Jansen & van der Klei, 2019). Clearly, an increase in mutation rate occurs when a deletion of *PEX19* is combined with some yet‐to‐be‐determined aspect of the aged phenotype, but whether peroxisomes are directly involved or not is unclear, and additional studies will be required to elucidate the mechanism behind Pex19‐dependent suppression of mutations in aged cells.

It has long been thought that the accumulation of mutations promotes aging. Many yeast DNA repair mutants have increased rates of mutation and decreased replicative lifespans (Table S5). However, the reduced lifespan in these mutants is likely due to an age‐independent decrease in cell viability (i.e., both mother and daughter cells have an increased rate of mortality). A previous study found that mutations did increase with yeast replicative age, but their low numbers (less than one per lifespan) indicate that mutations are not causing aging in wild‐type yeast (Kaya, Lobanov, & Gladyshev, 2015). Our findings support this conclusion because the deletion of *PEX19* has been reported to extend, not shorten, replicative lifespan (McCormick et al., 2015).

The human orthologs of *PEX19* and *PEX3*, together with other peroxins, are mutated in Zellweger syndrome, a severe cerebro‐hepato‐renal peroxisome biogenesis disorder (Fujiki, Yagita, & Matsuzaki, 2012). Given the evolutionary conservation of peroxisome biogenesis factors, it would be interesting to test the contribution of these genes in genome maintenance during mammalian cell aging and cancer development.

## 4 EXPERIMENTAL PROCEDURES

### 4.1 Yeast strains and growth conditions

Standard yeast media and growth conditions were used (Sherman, 2002). All yeast strains used in this study are derivatives of the BY4741 genetic background and are listed in Table S4.

### 4.2 High‐throughput replica‐pinning screen

High‐throughput manipulation of high‐density yeast arrays was performed with the RoToR‐HDA pinning robot (Singer Instruments). The MEP was introduced into the *MAT* **a** yeast deletion collection (EUROSCARF) through SGA methodology (Tong & Boone, 2006) using the DNY34 query strain. The procedure was performed twice in parallel to generate two independent sets of MEP yeast deletion arrays in 384‐colony format. When a specific MEP mutant was missing in one of the arrays, it was manually pinned over from the other set. Positions that were empty in both sets were filled with *his3*Δ*::kanMX* control strains, unless they were kept empty for plate identification purposes. Colonies from the two sets of MEP yeast deletion arrays were pinned onto YPD + G418 plates and incubated for 6 hr at 30°C. Each plate of each set was then pinned onto nine YPD plates containing 1 μM estradiol (18 replicates in total). At this step, colonies were pinned in 384 format using 1536 format pads, so that a smaller number of cells was deposited to prevent nutrient limitation. Plates were incubated for 7 days at 30°C and then scanned with a flatbed scanner. Subsequently, each plate was pinned onto one YPD plate containing 1 μM estradiol and incubated for 2 days at 30°C before scanning (“escaper test”). Colony area measurement was performed using the ImageJ software package (Schneider, Rasband, & Eliceiri, 2012) and the ImageJ plugin ScreenMill Colony Measurement Engine (Dittmar, Reid, & Rothstein, 2010), to assess colony circularity and size in pixels. The data were filtered to exclude artifacts by requiring a colony circularity score greater than 0.8. Colonies with a pixel area greater than 200 were considered escapers, and for each deletion strain, the ratio of escapers to total colonies in replica‐pinning experiments was used as the escaper frequency score.

### 4.3 Screen validation pipeline

Putative hits were initially analyzed by fluctuation test to measure the forward mutation rate at the endogenous *CAN1* locus. To do so, we used the strains from the YKO collection, because the strains from the MEP‐YKO collection are *can1Δ*. At first, we performed one fluctuation test per strain. If the mutation rate was higher than 1.5‐fold of the wild‐type mutation rate, the test was repeated another two or three times.

For all the genes whose deletion does not cause an increase in the mutation rate at the *CAN1* locus, the corresponding knockout strains from the MEP‐YKO collection were analyzed by fluctuation test to measure the escaper formation rate in young cells and the escaper formation frequency in replicatively aged cells. At first, we performed one fluctuation test per strain. If the escaper formation rate was higher than 1.5‐fold of the wild‐type escaper formation rate, the test was repeated another two or three times. In case no increase in escaper formation rate was detected but elevated age‐dependent escaper formation frequencies were observed, the experiment was repeated another one or two times. To report the “general mutation suppression genes” and the “MEP‐specific mutation suppression genes” (Tables 1 and 2), we chose a slightly more stringent cutoff (1.8‐fold), since this cutoff yielded more statistically supported enrichment of GO categories. Strains that consistently displayed an elevated age‐dependent escaper formation frequency were further validated by construction of a new knockout strain in a MEP *CAN1* background and by direct measurement of spontaneous mutation frequencies at the *CAN1* locus in young and aged cells. Each knockout strain was tested once. If the age‐dependent mutation frequencies were not higher than the wild‐type control, the strain was discarded as a false positive; if the age‐dependent mutation frequencies were increased compared with the wild‐type control, the experiment was repeated three times.

The identity of all validated strains from the YKO and MEP‐YKO collections was confirmed by barcode sequencing, as previously described (Mcmahon et al., 2011).

### 4.4 Measurements of the spontaneous forward mutation rate at the *CAN1* locus

Single colonies were inoculated in 5 ml YPD and grown up to saturation (2 days at 30°C). 100 μl was plated onto canavanine‐containing *SD* medium (50 μg/ml) to identify forward mutations in *CAN1*, and 50 μl of a 10^5^‐fold dilution was plated onto *SD* medium to count viable cells. Colonies were counted after 2 days of growth at 30°C, and the spontaneous forward mutation rate at the *CAN1* locus was determined by fluctuation test from nine independent cultures using the method of the median (Foster, 2006). Values represent the average of at least three independent experiments.

### 4.5 Measurements of spontaneous escaper formation rate and age‐dependent escaper formation frequencies

Single colonies were inoculated in 5 ml YPD and grown up to saturation (2 days at 30°C). 50 μl of a 50‐fold dilution (or a higher dilution, when needed) was plated onto YPD plates containing 1 μM estradiol to identify escaper occurrence in young cells (“young plates”), 50 μl of a 500‐fold dilution (or a higher dilution, when needed) was plated onto YPD plates containing 1 μM estradiol to identify escapers occurrence in aging cells (“old plates”), and 50 μl of a 500,000‐fold dilution was plated onto YPD plates to count viable cell number. Colonies were counted after 2 days of growth at 30°C (for “young plates” and YPD plates) or after two and after 7 days of growth at 30°C (for “old plates”). In “young plates,” colonies that were smaller than the colonies growing on the corresponding YPD plate were not counted, because for those colonies the escaper‐causing mutation occurred after plating. The spontaneous escaper formation rate in young cells was determined by fluctuation test from seven to 10 independent cultures using the MSS‐maximum‐likelihood estimator method from the FALCOR fluctuation analysis calculator (Hall, Ma, Liang, & Singh, 2009). Values represent the average of at least three independent experiments. Age‐dependent escaper formation frequencies were calculated by dividing the number of escaper colonies that appeared between day 2 and day 7 (on “old plates”) by the number of viable cells plated (determined from the YPD plates).

### 4.6 Measurements of spontaneous mutation frequencies at the *CAN1* locus in young and aged cells

Isolation of young and aged cells was performed essentially as previously described (Lindstrom & Gottschling, 2009) (see Supporting Information for details). Young (2 hr) and aged (20 hr) cells were plated on *SD* medium to assess cell viability and on canavanine‐containing *SD* medium (50 μg/ml) to identify forward mutations in *CAN1*. Colonies were counted after 2 days of growth at 30°C, and the spontaneous forward mutation frequencies at the *CAN1* locus were determined. Expected mutation frequencies in aged cells were calculated as previously described (Patterson & Maxwell, 2014).

### 4.7 Bud scar detection and counting

Purified mother cells (see above) were stained with propidium iodide (PI) (Sigma) to identify viable cells and with Calcofluor White (Fluorescent Brightener 28, Sigma) to detect bud scars. Bud scars from at least 50 PI‐negative cells (which were alive after magnetic sorting) were manually counted for each sample to determine the cells’ replicative age. See Supporting Information for details.

### 4.8 Gene Ontology enrichment analysis and functional annotation

GO enrichment analysis was performed with DAVID 6.8 (https://david.ncifcrf.gov/home.jsp) using the Functional Annotation tool (Huang, Sherman, & Lempicki, 2009). To reduce functional redundancy among GO terms, we used the REVIGO Web server (http://revigo.irb.hr/) with a cutoff value C = 0.5 (Supek, Bošnjak, Škunca, & Šmuc, 2011). Functional enrichment within the yeast global genetic similarity network was performed and visualized with TheCellMap.org (http://thecellmap.org/).

### 4.9 DNA damage sensitivity of young and aged cells

DNA damage sensitivity of young and aged wt and *pex19∆* cells was assessed as previously described (Novarina et al., 2017). See Supporting Information for details.

## CONFLICT OF INTEREST

None declared.

## AUTHORS’ CONTRIBUTION

DN and MC designed the study. DN, GEJ, KB, TS, NCvO, HGK, and MC performed research and data analysis. DN and MC wrote the manuscript. DN, GEJ, KB, HGK, LMV, and MC reviewed and edited the manuscript.

## Data Availability

The data that support the findings of this study are available from the corresponding author upon reasonable request.
