# Supplementary material for: A genome‐wide screen identifies genes that suppress the accumulation of spontaneous mutations in young and aged yeast cells
Source: Aging Cell. 2019 Dec 18;19(2):e13084. doi: 10.1111/acel.13084 (PMC6996960; doi:10.1111/acel.13084)
Supplement: Supplementary file 3 [file ACEL-19-e13084-s003.pdf]

Supplemental Experimental Procedures

Measurements of spontaneous mutation frequencies at the *CAN1* locus in young and aged cells

Isolation of young and aged cells was performed essentially as previously described (Janssens et al., 2015; Lindstrom & Gottschling, 2009). 1.5 x 10^9 cells from a log-phase MEP culture were washed with cold phosphate buffered saline (PBS), resuspended in cold PBS containing 7 mg/ml Sulfo-NHS-LC-Biotin (Thermo Scientific) and incubated for 20 min at room temperature with gentle shaking. Biotinylated cells were then washed with PBS, resuspended in 250 ml of pre-warmed YPD medium and allowed to recover for 2 h at 30°C with shaking. Estradiol was added to a final concentration of 1 µM to induce the MEP (aging starts here). After 2 h of incubation at 30°C with shaking, 100 ml were harvested (young cells), while the rest of the culture (150 ml) was inoculated in a total volume of 1 L YPD containing 1 µM estradiol and 100 µg/ml ampicillin (to discourage bacterial contamination), and incubated at 30°C with shaking. Young cells were washed with cold PBS, resuspended in 5 ml cold PBS and incubated with 100 µl streptavidin-coated BioMag beads (Qiagen) in a 5 ml LoBind tube (Eppendorf) at 4°C with gentle shaking for 30 min. Cells were gently pelleted at 4°C (3 min 1800 × g), resuspended in 7 ml cold YPD and transferred to a glass test tube (Lab Logistics Group). The tube was placed in a magnet ("The Big Easy" EasySep Magnet, Stemcell Technologies) for 5 min on ice. Cells were then washed three times by removing supernatant by pipetting, resuspending them in 7 ml cold YPD and incubating for 5 min on ice in the magnet. Finally, cells were resuspended in 5.2 ml PBS and transferred in a 5 ml LoBind tube (Eppendorf). Of the 5.2 ml of purified mother cells, 100 µl were stained for bud scars counting (see below); 100 µl were diluted 1000x and plated on SD medium to assess cell viability; the remaining 5 ml were pelleted and plated on canavanine-containing SD medium (50 µg/ml) to identify forward mutations in *CAN1*. After 20 h of MEP induction, the entire

aged 1 L culture was harvested. The aged cells were processed similarly to the young cells, with slight modifications because of the higher number of cells due to the presence of daughter cells. For beading, the cells were split into 4 different 5 ml LoBind tubes, and 50 µl streptavidin coated BioMag beads were added to each tube. For magnetic sorting, two glass tubes were used and cells were washed four times.

For both young and aged samples, colonies were counted after 2 d of growth at 30°C and the spontaneous forward mutation frequencies at the *CAN1* locus were determined. Expected mutation frequencies in aged cells were calculated as previously described (Patterson & Maxwell, 2014).

Bud scar detection and counting

Purified mother cells (see above) were stained with propidium iodide (PI) (Sigma) to identify viable cells and with Calcofluor White (Fluorescent Brightener 28, Sigma) to detect bud scars. 100 µl of purified mother cells in PBS (~5 x 10^5 cells) were stained with 2 µl of a 2 mM PI (Sigma) solution for 30 min at 30°C. Cells were then washed with ddH_2O, fixed in 500 µl of 3.7% formaldehyde for 30 min at room temperature, washed with PBS, resuspended in 100 µl PBS and stored at 4°C. Just before imaging, cells were stained with Calcofluor White for 5 min at room temperature, washed with PBS and resuspended in 5-10 µl PBS. Images were acquired using a DeltaVision Elite imaging system (Applied Precision (GE), Issaquah, WA, USA) composed of an inverted microscope (IX-71; Olympus) equipped with a Plan Apo 100X oil immersion objective with 1.4 NA, InsightSSITM Solid State Illumination, excitation and emission filters for DAPI and A594, ultimate focus and a CoolSNAP HQ2 camera (Photometrics, Tucson, AZ, USA). Stacks of 30 images with 0.2 µm spacing were taken at an exposure time of 5 ms at 10% intensity for DAPI (Calcofluor White staining) and 50 ms at 32% intensity for A594 (PI staining). Reference bright-field images were also taken.

Fluorescent images were subjected to 3D deconvolution using SoftWoRx 5.5 software (Applied Precision). Processing of all images was performed using Fiji (ImageJ, National Institute of Health) (Schneider et al., 2012). Bud scars from at least 50 PI-negative cells (which were alive after magnetic sorting) were manually counted for each sample to determine the cells' replicative age.

DNA damage sensitivity of young and aged cells

DNA damage sensitivity of young and aged wt and *pex19*Δ cells was assessed as described in (Novarina et al., 2017). Exponentially growing cultures were diluted to a concentration of 4×10^4 cells/ml and estradiol was added to a final concentration of 1 μM to induce the MEP. Half of the culture was treated 2 h after induction by estradiol (young cells), while the other half was incubated for 20 h shaking at 30 °C (aged cells). Young and aged cells were mock-treated or treated with H_2O_2 or MMS at the indicated doses. Cells were diluted tenfold prior to plating on four plates (technical replicates) per dose/time point. Plating volumes were adjusted for young and aged cells to obtain ~100 colony forming units per plate. Mock-treated aged cells were also plated on control plates containing 1 μM estradiol to detect escapers. If the escaper frequency in the culture was higher than 10%, the experiment was discarded.

References

Janssens, G. E., Meinema, A. C., González, J., Wolters, J. C., Schmidt, A., Guryev, V., … Heinemann, M. (2015). Protein biogenesis machinery is a driver of replicative aging in yeast. *ELife*, *4*(September 2015), e08527. https://doi.org/10.7554/eLife.08527

Lindstrom, D. L., & Gottschling, D. E. (2009). The mother enrichment program: a genetic system for facile replicative life span analysis in Saccharomyces cerevisiae. *Genetics*, *183*(2), 413–422. https://doi.org/10.1534/genetics.109.106229

Novarina, D., Mavrova, S. N., Janssens, G. E., Rempel, I. L., Veenhoff, L. M., & Chang, M.

(2017). Increased genome instability is not accompanied by sensitivity to DNA damaging agents in aged yeast cells. *DNA Repair*, *54*, 1–7. https://doi.org/10.1016/j.dnarep.2017.03.005

Patterson, M. N., & Maxwell, P. H. (2014). Combining magnetic sorting of mother cells and fluctuation tests to analyze genome instability during mitotic cell aging in Saccharomyces cerevisiae. *Journal of Visualized Experiments*, *92*, e51850. https://doi.org/10.3791/51850

Schneider, C. A., Rasband, W. S., & Eliceiri, K. W. (2012). NIH Image to ImageJ : 25 years of image analysis. *Nature Methods*, *9*(7), 671–675. https://doi.org/10.1038/nmeth.2089

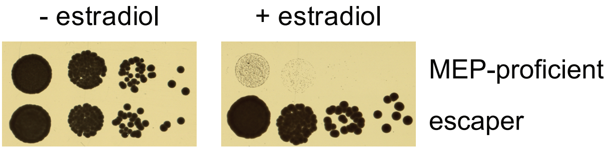

Figure S1. Exclusion of strains that escaped before the beginning of the screen.
When serial dilutions of strains from the MEP-YKO collection are spotted in the presence of estradiol, growth of MEP-proficient strains is restricted, while escaper strains grow normally. An example of one MEP-proficient strain and one escaper is shown.

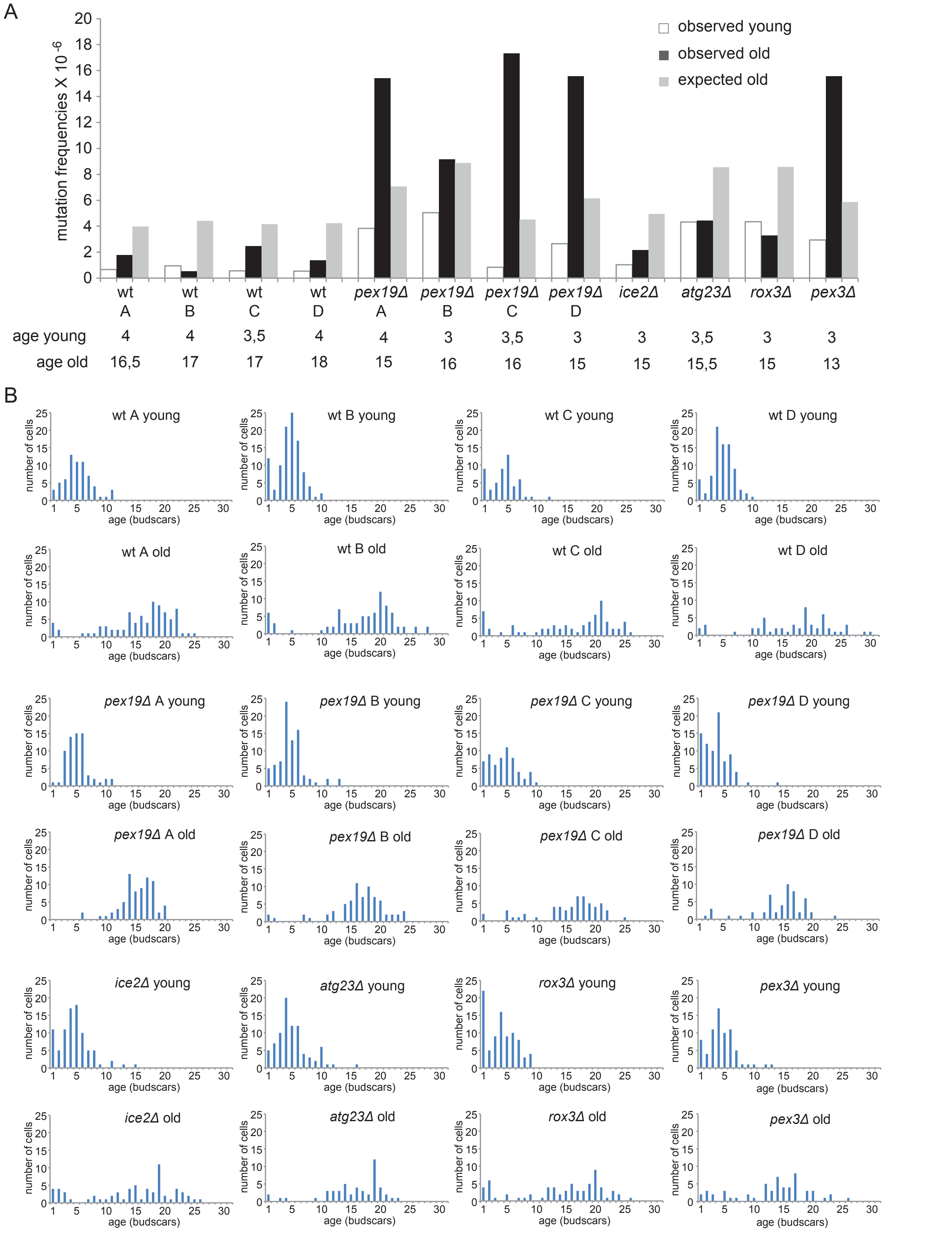

Figure S2. Raw data for all the age-dependent mutation frequency measurement experiments. (**A**) Mutation frequencies at the *CAN1* locus in young and old cells from the indicated strains. In the case of old cells, both observed and expected mutation frequencies are shown. For each individual experiment, the median replicative age of the young and old cell populations is indicated. (**B**) Bud scars distribution of young and old cell populations from each experiment.

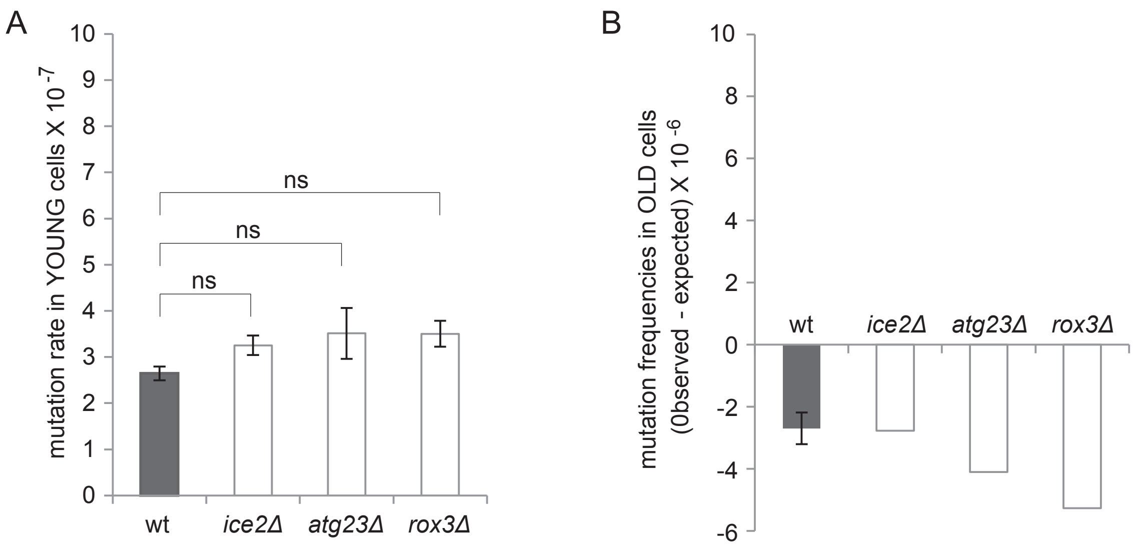

Figure S3. *ICE2*, *ATG23*, and *ROX3* did not validate as age-specific mutation suppression genes. (**A**) *CAN1* forward mutation rate in young wt, *ice2Δ*, *atg23Δ*, and *rox3Δ* cells. Mean values from three independent experiments are plotted. Error bars represent standard error. ns: non-significant. (**B**) Age-dependent mutation frequencies at the *CAN1* locus in wt (replicative age ~17), *ice2Δ* (replicative age ~15), *atg23Δ* (replicative age ~15.5), and *rox3Δ* (replicative age ~15) cells. The difference between observed and expected mutation frequency is plotted. For the wt, the mean value from four independent experiments is plotted. Error bars represent standard error. For the mutants, only one experiment is shown (see the Results and Material and Methods sections for details).

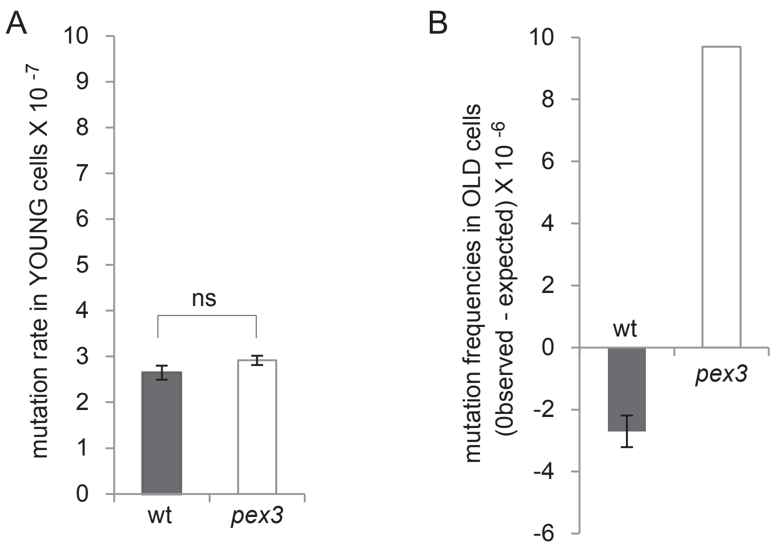

Figure S4. *PEX3* deletion results in elevated spontaneous mutations in aged cells.
(**A**) *CAN1* forward mutation rate in young wt, and *pex3Δ* cells. Mean values from three or four independent experiments are plotted. Error bars represent standard error. ns: non-significant.
(**B**) Age-dependent mutation frequencies at the *CAN1* locus in wt (replicative age ~17) and *pex3Δ* (replicative age ~13) cells. The difference between observed and expected mutation frequency is plotted. For the wt, the mean value from four independent experiments is plotted. Error bars represent standard error. For *pex3Δ*, only one experiment is shown.

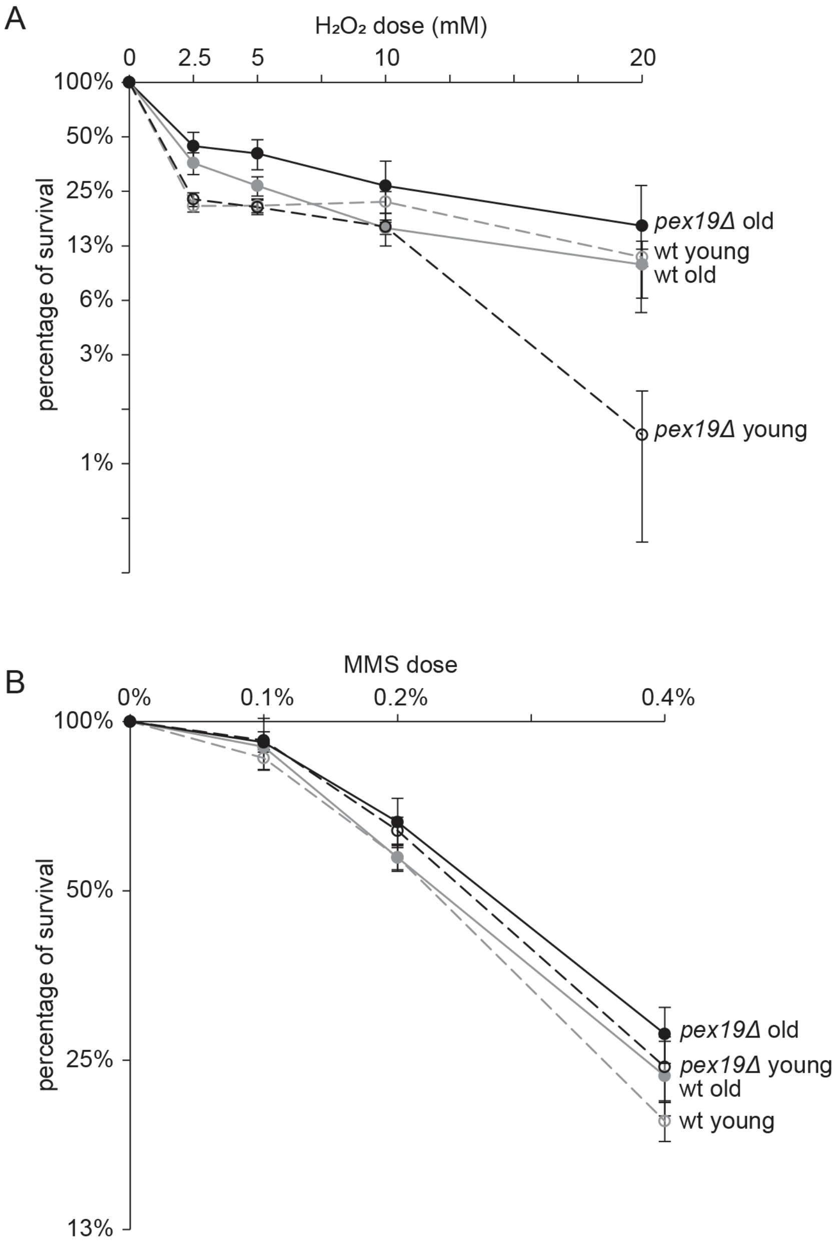

Figure S5. Aged *pex19Δ* cells do not display increased sensitivity to H_2O_2 or MMS.
Survival curves for young (t = 2 h) and aged (t = 20 h, corresponding to age ~13) wt and *pex19Δ* MEP cells exposed to H_2O_2 (**A**) or MMS (**B**) at the indicated doses. The duration of the H2O2 and MMS treatments were 30 min and 20 min, respectively. Mean values from at least three independent experiments are plotted. Error bars represent standard error.

Table S1. Ranked list of top overlapping screens (ScreenTroll)

#	Screen	ORFs in screen	common hits	Rank Score	Ref	overlapping genes
1	Mutator phenotype at *CAN1* gene	33	12	2.29×10^{-13}	Huang et al. 2003. PNAS 100; 11529-11534	PMS1, MSH2, SHU2, MSH6, MME1, OGG1, RAD27, RAD57, PSY3, MLH1, RAD54, RAD55
2	4-nitroquinoline-1-oxide sensitive (all statistically significant hits)	25	7	2.35×10^{-7}	Lee et al. 2005. Plos Genetics 1; e24	MPH1, RAD10, RAD57, SNF2, RAD54, RAD55, RAD2
3	Hydroxyurea Sensitive	133	13	8.40×10^{-7}	Parsons et al. 2004. Nature Biotech 22; 62-69	SNF5, MME1, OPI9, NAT3, RAD57, SAC3, SNF2, ROX3, MET18, NUP84, RAD54, RAD55, VMA6
4	Cisplatin Sensitive (top 50)	50	8	3.15×10^{-6}	Wu et al. 2004. Cancer Res 64; 3940-3948	MPH1, RAD10, SHU2, RAD57, PSY3, RAD54, RAD55, RAD2
5	Cisplatin sensitive (all statistically significant hits)	67	9	3.35×10^{-6}	Lee et al. 2005. Plos Genetics 1; e24	MPH1, RAD10, SHU2, RAD57, PSY3, RAD54, RAD55, RAD2, GPM2
6	MMS sensitive (all statistically significant hits)	25	6	4.89×10^{-6}	Lee et al. 2005. Plos Genetics 1; e24	MPH1, SHU2, RAD57, PSY3, RAD54, RAD55
7	Synthetic lethal/sick with ubc9-2	313	18	1.42×10^{-5}	Makhnevych et al. 2009. Molecular Cell 33; 124-135	MFT1, ASM4, RAD57, RAD27, SEM1, SAC3, NIP100, MET18, UBP1, THP2, SPT8, RAD54, YPL205C, RLF2, RAD55, SPT3, NMD2, YNL140C
8	MMS Sensitive	103	10	1.83×10^{-5}	Chang et al PNAS 2002. 99;16934-16939	RAD57, RAD27, NAT3, PSY3, MET18, NUP84, LDB7, RAD54, RAD55, SPT4
9	UV Sensitive Strains	31	6	1.86×10^{-5}	Birrell et al. 2001. PNAS 98; 12608-12613	RAD10, RAD57, SNF2, RAD54, RAD55, RAD2
10	Most (SSS) gamma sensitive	31	6	1.86×10^{-5}	Bennett et al. 2001. Nature Genetics 29; 436-434	NUP120, NAT3, RAD57, RSC1, NUP84, RAD55
11	Synthetic lethal/sick with ulp1-333sgg	332	18	3.16×10^{-5}	Makhnevych et al. 2009. Molecular Cell 33; 124-135	MFT1, ASM4, RAD57, RAD27, SEM1, SAC3, NIP100, DSS4, MET18, THP2, SPT8, RAD54, YPL205C, RLF2, RAD55, SPT3, YNL140C, LRS4
12	Slow growing diploid strains on YPD	489	22	6.64×10^{-5}	Giaever et al. 2002. Nature. 418; 387-391	NUP120, RPP1A, SNF5, SRP40, OPI9, RAD57, RAD27, NAT3, SAC3, SEM1, SNF2, MET18, ROX3, THP2, RSC1, NUP84, RPL13B, RAD54, RAD55, SPT4, RPL7A, VMA6
13	Caffeine Sensitive	174	12	8.18×10^{-5}	Parsons et al. 2004. Nature Biotech 22; 62-69	MFT1, SNF5, THP1, OPI9, NAT3, SAC3, SEM1, YLR358C, THP2, RPL13B, LDB7, SPT4
14	Most (+++) MMS Sensitive	78	8	8.95×10^{-5}	Chang et al PNAS 2002. 99;16934-16939	RAD54, RAD55, NAT3, RAD27, RAD57, PSY3, MET18, NUP84
15	2-dimethylaminoethyl chloride sensitive (all statistically significant hits)	60	7	1.11×10^{-4}	Lee et al. 2005. Plos Genetics 1; e24	MPH1, SHU2, RAD27, RAD57, PSY3, RAD54, RAD55
16	Chromosome instability (CIN) genes	130	10	1.36×10^{-4}	Yuen et al. 2007 PNAS 104; 3925-3930	MFT1, RAD10, NUP120, THP1, RAD57, RAD27, THP2, RAD54, RAD55, YNL140C
17	Synthetic lethal/sick with siz1 siz2 double null	238	13	4.26×10^{-4}	Makhnevych et al. 2009. Molecular Cell 33; 124-135	MFT1, ASM4, RAD57, RAD27, NIP100, SEM1, SAC3, SPT8, RAD54, RLF2, RAD55, SPT3, LRS4
18	Oxaliplatin sensitive (all statistically significant hits)	36	5	4.94×10^{-4}	Lee et al. 2005. Plos Genetics 1; e24	RAD10, RAD57, RAD54, RAD55, RAD2
19	Camptothecin Sensitive	82	7	7.82×10^{-4}	Parsons et al. 2004. Nature Biotech 22; 62-69	MME1, NAT3, RAD57, SAC3, NUP84, RAD54, RAD55
20	Increased LOH in at least one assay	61	6	8.91×10^{-4}	Andersen et al. 2008. Genetics 179; 1179-1195	RAD27, NUP84, RAD54, RLF2, LRS4, ICE2
21	Gamma sensitive	137	9	9.59×10^{-4}	Bennett et al. 2001. Nature Genetics 29; 436-434	RAD10, NUP120, NAT3, RAD27, RAD57, RSC1, NUP84, LDB7, RAD55
22	Sulfometuron methyl Sensitive	85	7	9.70×10^{-4}	Parsons et al. 2004. Nature Biotech 22; 62-69	SNF5, OPI9, NIP100, SNF2, SPT8, SPT3, VMA6

Table S1. ScreenTroll phenotypic enrichment analysis. ScreenTroll analysis identifies overlaps between published screens and our gene dataset (cutoff 75% escaper frequency, determined by CLIK). The top overlaps are related to genome instability and DNA damage sensitivity. “ORFs in screen” refers to the total number of hits identified in the overlapping screen.

Table S2. List of putative age-specific mutator genes

gene	function	young cells		old cells	
		***CAN1* mutation rate**	**escaper formation rate**	**age-dependent escaper frequency**	**validated at *CAN1***
ICE2	integral ER membrane protein	$3,3 \times 10^{-7}$	$3,5 \times 10^{-7}$	$2,6 \times 10^{-1}$	NO
PEX19	biogenesis of peroxisomes	$2,9 \times 10^{-7}$	$3,4 \times 10^{-7}$	$1,2 \times 10^{-1}$	YES
ATG23	autophagy and cytoplasm-to-vacuole targeting	$3,5 \times 10^{-7}$	$3,2 \times 10^{-7}$	$7,1 \times 10^{-3}$	NO
ROX3	RNA polymerase II subunit	$3,5 \times 10^{-7}$	$2,7 \times 10^{-7}$	$4,9 \times 10^{-3}$	NO
wild type		$2,6 \times 10^{-7}$	$2,9 \times 10^{-7}$	$7,7 \times 10^{-4}$	

Table S3. Escaper formation is not always caused by mutations at the *cre-EBD78* locus.

escaper n.	co-segregation of escaper phenotype and *cre-EBD78* locus?
1	no
2	no
3	yes
4	no
5	no
6	yes
7	yes
8	yes
9	yes

Table S4. Yeast strains used in this study.

Strain name	Relevant genotype	Source
BY4741	*MAT***a** *his3Δ1 leu2Δ0 ura3Δ0 met15Δ0*	(Brachmann et al., 1998)
UCC8773	*MAT***a** *his3Δ1 leu2Δ0 ura3Δ0 lys2Δ0 hoΔ::Pscw11-cre-EBD78-natMX loxP-CDC20-intron-loxP-hphMX loxP-UBC9-loxP-LEU2*	(Henderson, Hughes, & Gottschling, 2014)
UCC8774	*MATα his3Δ1 leu2Δ0 ura3Δ0 trp1Δ63 hoΔ::Pscw11-cre-EBD78-natMX loxP-CDC20-intron-loxP-hphMX loxP-UBC9-loxP-LEU2*	(Henderson et al., 2014)
Y7092	*MATα his3Δ1 leu2Δ0 ura3Δ0 can1Δ::STE2pr-Sp_his5 lyp1Δ met15Δ0*	(Tong & Boone, 2007)
DNY34	*MATα his3Δ1 leu2Δ0 ura3Δ0 can1Δ::STE2pr-Sp_his5 lyp1Δ met15Δ0 hoΔ::PSCW11-cre-EBD78-natMX loxP-UBC9-loxP-LEU2 loxP-CDC20-Intron-loxP-hphMX*	This study[1]
DNY80	*MAT***a** *his3Δ1 leu2Δ0 ura3Δ0 lys2Δ0 met15Δ0 hoΔ::Pscw11-cre-EBD78-natMX loxP-CDC20-intron-loxP-hphMX loxP-UBC9-loxP-LEU2 ice2Δ::kanMX*	This study[1]
DNY99	*MAT***a** *his3Δ1 leu2Δ0 ura3Δ0 lys2Δ0 met15Δ0 hoΔ::Pscw11-cre-EBD78-natMX loxP-CDC20-intron-loxP-hphMX loxP-UBC9-loxP-LEU2 pex19Δ::kanMX*	This study[1]
DNY101	*MAT***a** *his3Δ1 leu2Δ0 ura3Δ0 lys2Δ0 met15Δ0 hoΔ::Pscw11-cre-EBD78-natMX loxP-CDC20-intron-loxP-hphMX loxP-UBC9-loxP-LEU2 rox3Δ::kanMX*	This study[1]
DNY102	*MAT***a** *his3Δ1 leu2Δ0 ura3Δ0 lys2Δ0 met15Δ0 hoΔ::Pscw11-cre-EBD78-natMX loxP-CDC20-intron-loxP-hphMX loxP-UBC9-loxP-LEU2 atg23Δ::kanMX*	This study[1]
DNY105	*MAT***a** *his3Δ1 leu2Δ0 ura3Δ0 lys2Δ0 met15Δ0 hoΔ::Pscw11-cre-EBD78-natMX loxP-CDC20-intron-loxP-hphMX loxP-UBC9-loxP-LEU2 pex3Δ::kanMX*	This study[1]

[1]DNY34 was obtained from Y7092 and UCC8773 by crossing and tetrad dissection. The *ice2Δ::kanMX* strain from the deletion collection (EUROSCARF) strain was crossed with strain UCC8774 by standard yeast genetics to create the strain DNY80. Strains DNY99, DNY101, DNY102 and DNY105 were constructed by standard PCR-mediated gene deletion in strain UCC8773.

References

Brachmann, C. B., Davies, A., Cost, G. J., Caputo, E., Li, J., Hieter, P., & Boeke, J. D. (1998). Designer deletion strains derived from *Saccharomyces cerevisiae* S288C: a useful set of strains and plasmids for PCR-mediated gene disruption and other applications. *Yeast*, *14*(2), 115–132. https://doi.org/10.1002/(SICI)1097-0061(19980130)14:2<115::AID-YEA204>3.0.CO;2-2

Henderson, K. A., Hughes, A. L., & Gottschling, D. E. (2014). Mother-daughter asymmetry of pH underlies aging and rejuvenation in yeast. *ELife*, *3*, e03504. https://doi.org/10.7554/eLife.03504

Tong, A. H. Y., & Boone, C. (2007). High-throughput strain construction and systematic synthetic lethal screening in *Saccharomyces cerevisiae*. *Methods in Microbiology*, *36*, 369–386, 706–707. https://doi.org/10.1016/S0580-9517(06)36016-3

Table S5. Replicative lifespan (RLS) of gene deletion mutants identified in this study.

Gene deleted	Mutation/escaper rate	Change in RLS	Source of RLS data
General mutation suppression genes			
RAD27	167.1	61.8% ↓	(Hoopes et al., 2002)
MME1	45.1	17.6% ↓	(Smith et al., 2008)
RAD57	37.2	43.2% ↓	(Park et al., 1999)
RAD55	35.8	59.5% ↓	(Managbanag et al., 2008)
VMA6	22.5	58.3% ↓	(Managbanag et al., 2008)
MEP-specific mutation suppression genes			
RPP1A	34.4	30.7% ↑	(McCormick et al., 2015)
RPL13B	26.3	36.6% ↑	(McCormick et al., 2015)
GTO3	7.6	21.3% ↑	(McCormick et al., 2015)
Age-dependent mutation suppression genes			
PEX19	N/A	16.3% ↑	(McCormick et al., 2015)

References

Hoopes, L. L. M., Budd, M., Choe, W., Weitao, T., & Campbell, J. L. (2002). Mutations in DNA replication genes reduce yeast life span. *Molecular and Cellular Biology*, *22*(12), 4136–4146. https://doi.org/10.1128/mcb.22.12.4136-4146.2002

Managbanag, J. R., Witten, T. M., Bonchev, D., Fox, L. A., Tsuchiya, M., Kennedy, B. K., & Kaeberlein, M. (2008). Shortest-path network analysis is a useful approach toward indentifying genetic determinants of longevity. *PLoS ONE*, *3*(11), e3802. https://doi.org/10.1371/journal.pone.0003802

McCormick, M. A., Delaney, J. R., Tsuchiya, M., Tsuchiyama, S., Shemorry, A., Sim, S., … Kennedy, B. K. (2015). A comprehensive analysis of replicative lifespan in 4,698 single-gene deletion strains uncovers conserved mechanisms of aging. *Cell Metabolism*, *22*(5), 895–906. https://doi.org/10.1016/j.cmet.2015.09.008

Park, P. U., Defossez, P.-A., & Guarente, L. (1999). Effects of mutations in DNA repair genes on formation of ribosomal DNA circles and life span in *Saccharomyces cerevisiae*. *Molecular and Cellular Biology*, *19*(5), 3848–3856. https://doi.org/10.1128/mcb.19.5.3848

Smith, E. D., Tsuchiya, M., Fox, L. A., Dang, N., Hu, D., Kerr, E. O., … Kennedy, B. K. (2008). Quantitative evidence for conserved longevity pathways between divergent eukaryotic species. *Genome Research*, *18*(4), 564–570. https://doi.org/10.1101/gr.074724.107
