# Supplementary material for: A genome‐wide screen identifies genes that suppress the accumulation of spontaneous mutations in young and aged yeast cells
Source: Aging Cell. 2019 Dec 18;19(2):e13084. doi: 10.1111/acel.13084 (PMC6996960; doi:10.1111/acel.13084)
Supplement: Supplementary file 4 [file ACEL-19-e13084-s004.docx]

**File S1. Complete escaper frequency data from the screen and validation of mutation suppression genes.**

**File S2. SAFE enrichments and GO enrichment analysis for general and MEP-specific mutation suppression genes.**
